# Supplementary material for: Chamber-specific chromatin architecture guides functional interpretation of disease-associated Cis-regulatory elements in human cardiomyocytes
Source: Nat Commun. 2026 Jan 12;17:117. doi: 10.1038/s41467-025-67220-7 (PMC12796357; doi:10.1038/s41467-025-67220-7)
Supplement: Supplementary file 1 — Supplementary Information [file 41467_2025_67220_MOESM1_ESM.pdf]

Supplementary Figures

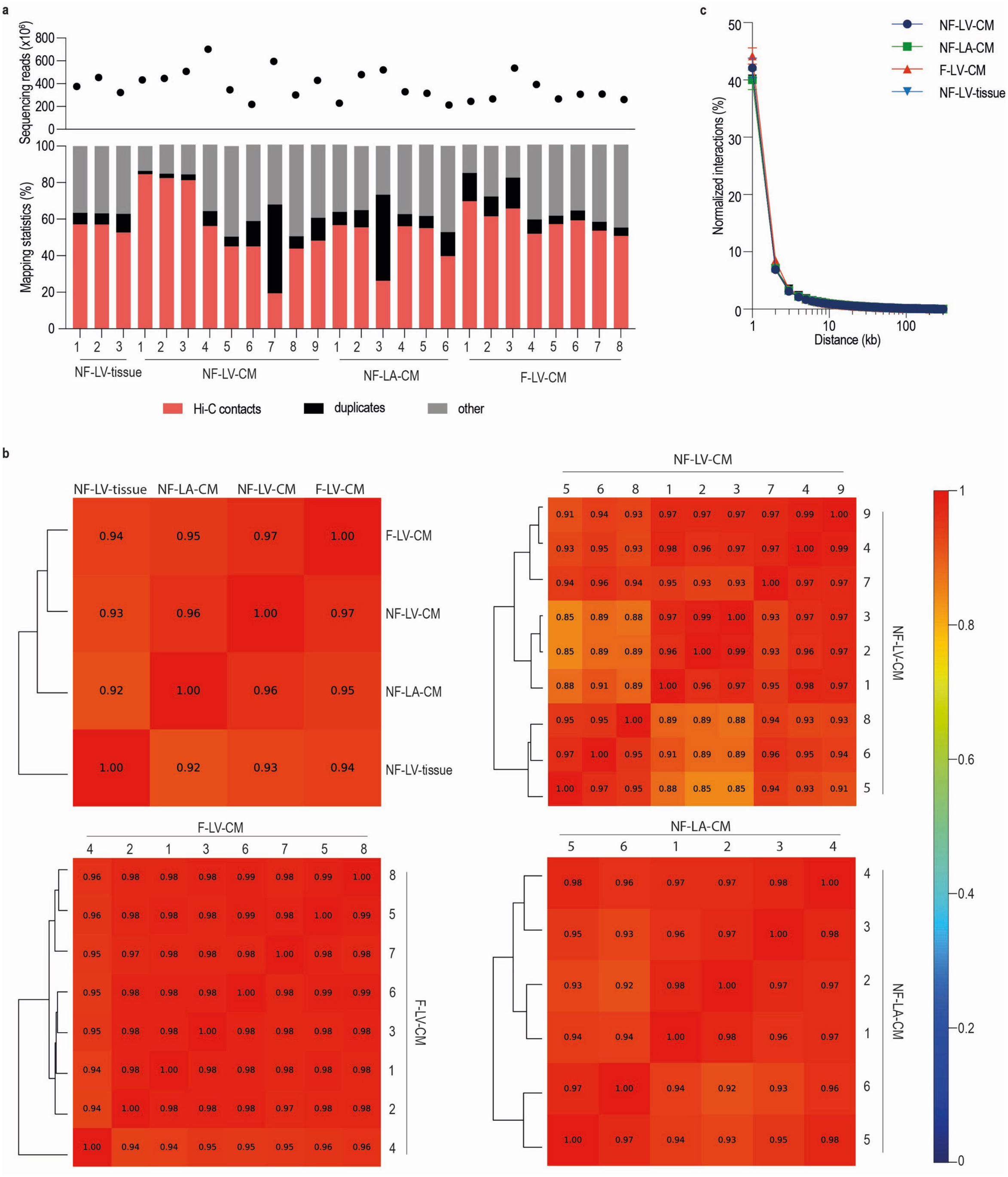

Supplementary Fig.1: Quality and correlation of Hi-C sequencing reads obtained from different biological replicates.
a Sequencing and mapping statistics of Hi-C data.
b Heatmaps of Spearman correlation coefficients of genome-wide Hi-C reads for the different biological replicates at 100 kb resolution.
c Line plot of genomic distance versus contact counts using 100 kb resolution Hi-C matrices.

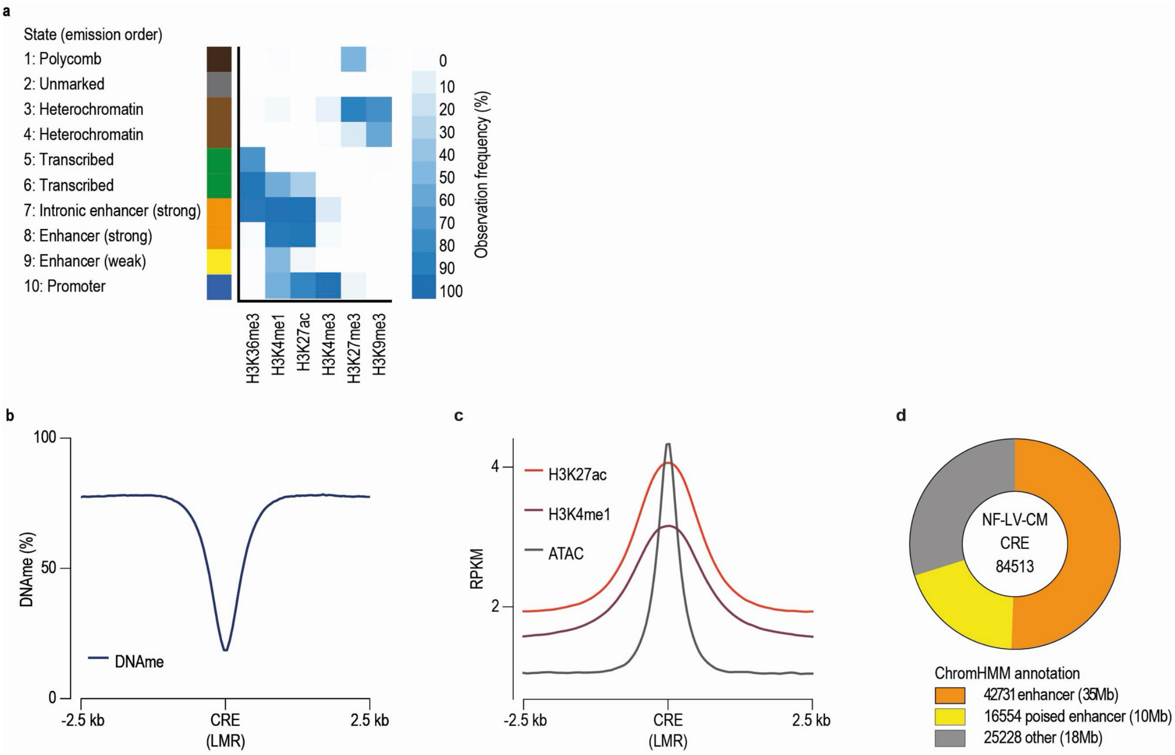

Supplementary Fig. 2: Chromatin state of CREs.
a Chromatin states were learned using a multi-variant hidden Markov model based on genome-wide occurrence of combinations of different histone marks (ChromHMM). Shown is a frequency heatmap.
b,c Profiles of CpG methylation (**b**, %), histone modifications, and chromatin accessibility (**c**, enrichment) flanking the CRE centers of NF-LV-CM.
d *Cis*-regulatory elements (CREs; Low-methylated regions, LMRs) detected in NF-LV-CM were categorized into enhancers, poised enhancers, and other chromatin states according to the ChromHMM model (see **a**). Shown are the number of CREs for each category, and the genomic length is annotated.

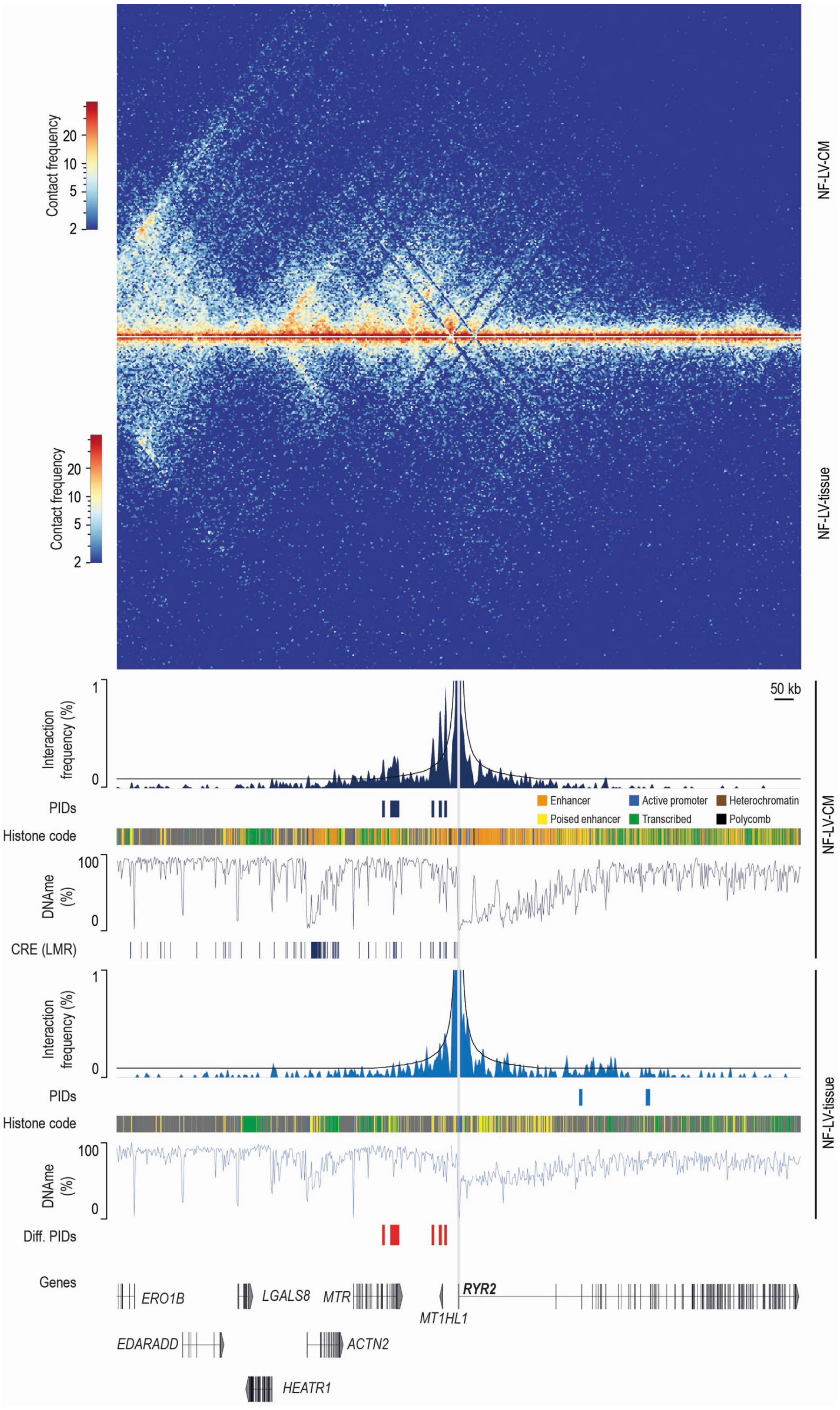

Supplementary Fig. 3: Comparison of cardiac tissue and CM chromatin interactions for the *RYR2* locus. Original traces of chromatin interaction frequency heatmap at 5 kb resolution (Hi-C) and histone marks (ChromHMM), virtual viewpoint analysis of *RYR2* promoter interactions (Hi-C), and mCpG (DNA methylation sequencing). *Cis*-regulatory elements (CREs; Low-methylated regions, LMRs), PIDs, and differential PIDs are annotated. Hi-C data derived from *n* biological replicates: NF-LV-CM, 3; NF-LV-CN, 3. Diff. PIDs, Differential promoter-interacting domains.

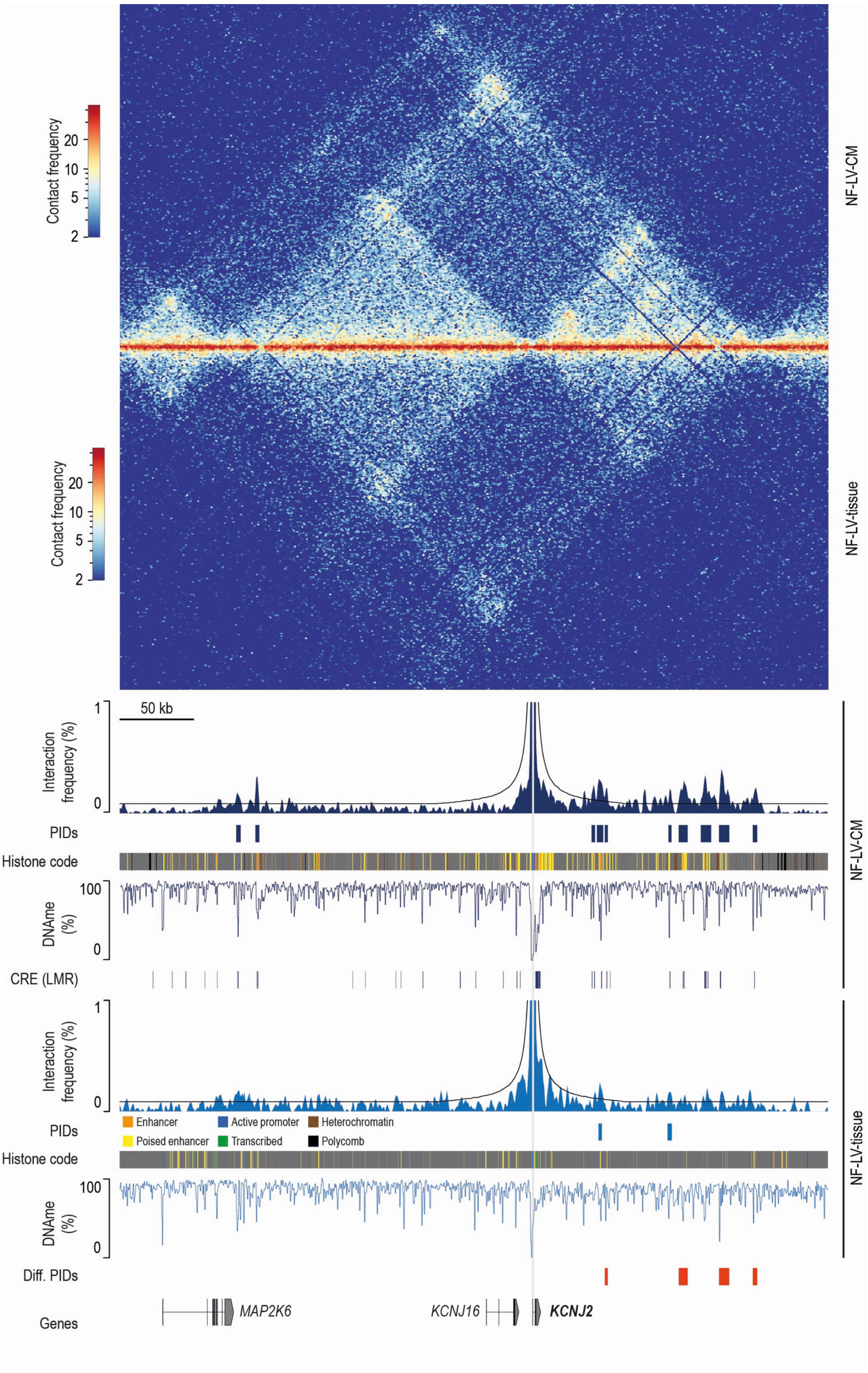

Supplementary Fig. 4: Comparison of cardiac and CM chromatin interactions for the *KCNJ2* locus.
see legend Supplementary Fig. 3

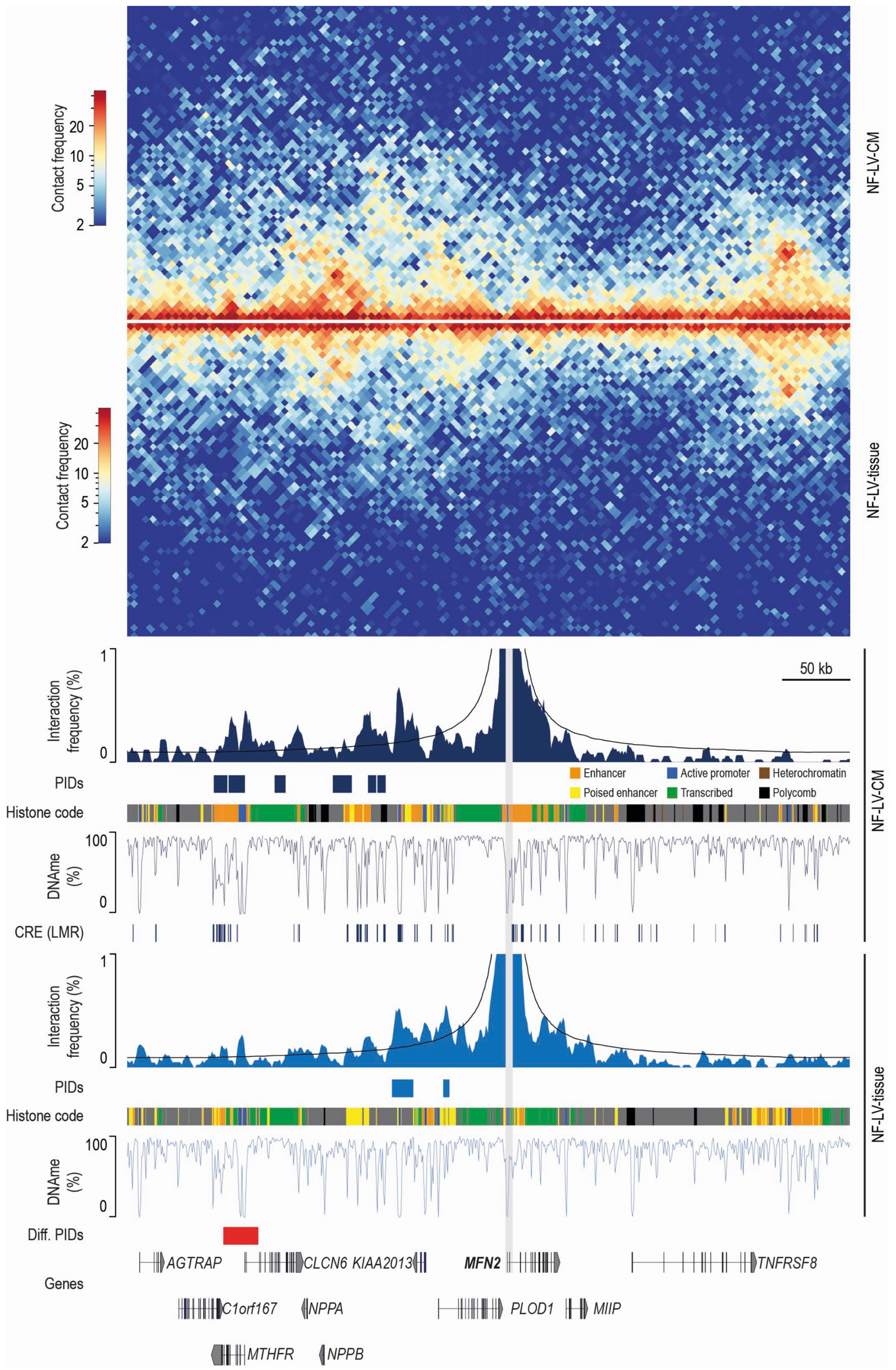

Supplementary Fig. 5: Comparison of cardiac and CM chromatin interactions for the *MFN2* locus.
see legend Supplementary Fig. 3

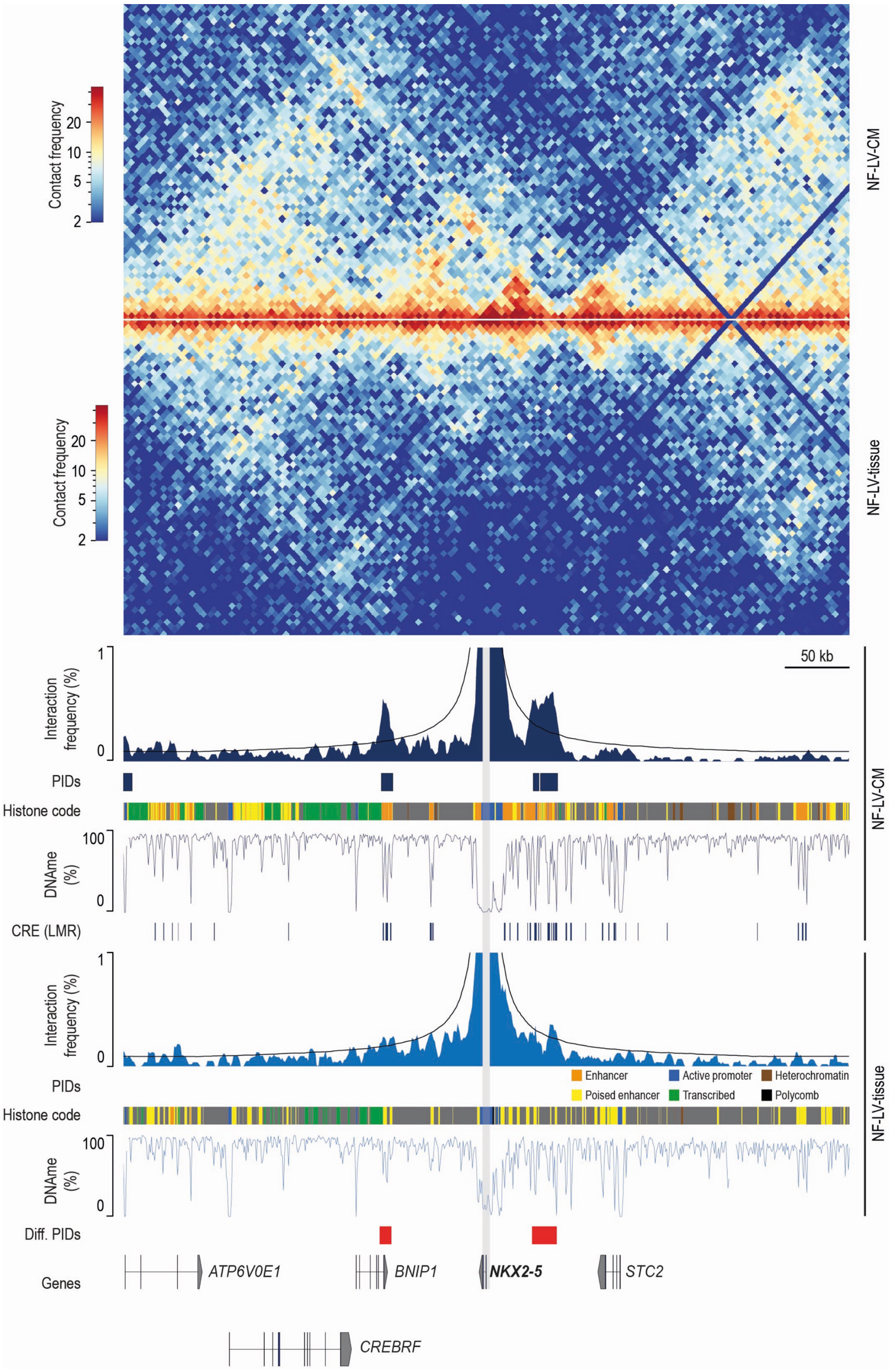

Supplementary Fig. 6: Comparison of cardiac and CM chromatin interactions for the *NKX2-5* locus.
see legend Supplementary Fig. 3

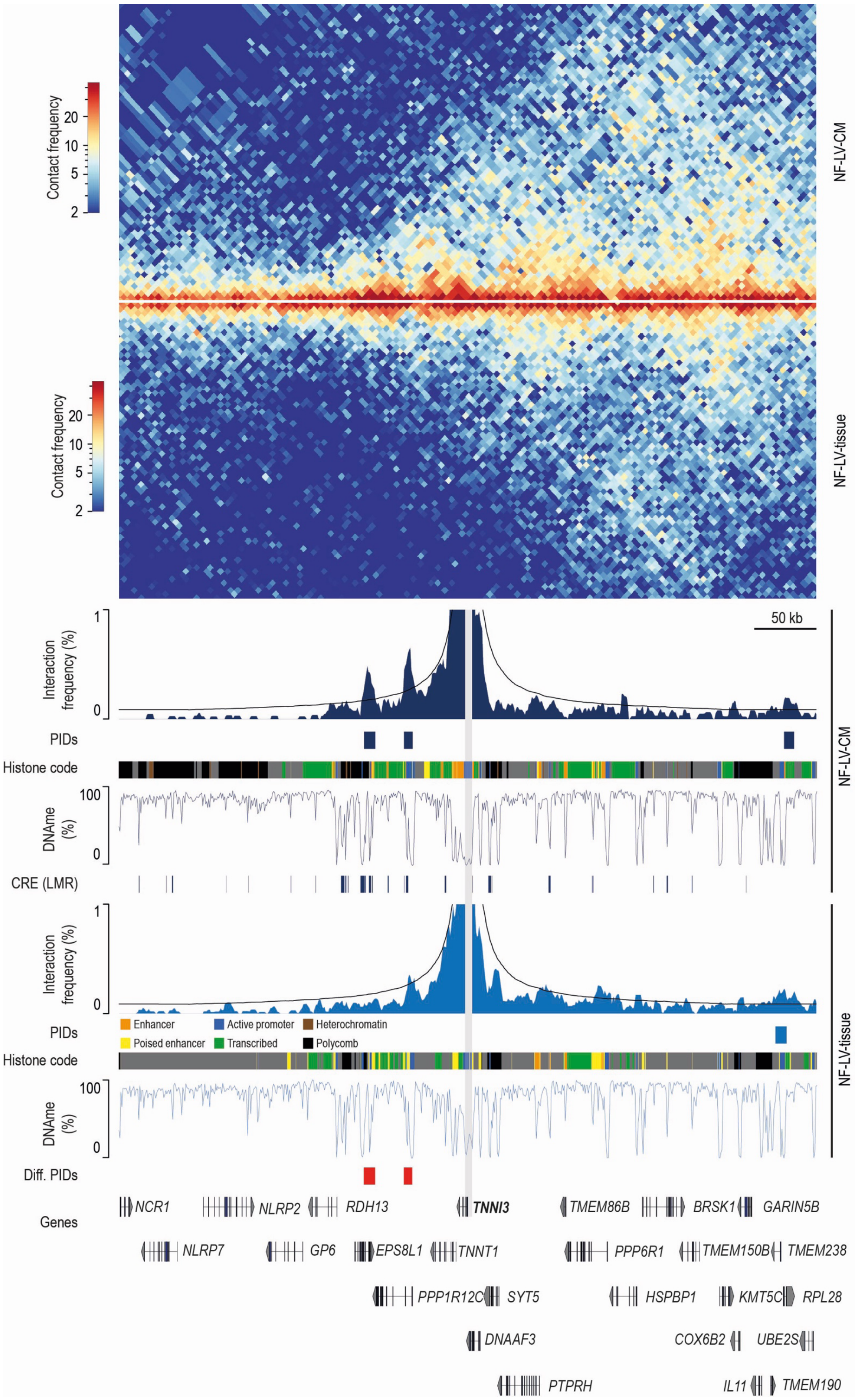

Supplementary Fig. 7: Comparison of cardiac and CM chromatin interactions for the *TNNI3* locus.
see legend Supplementary Fig. 3

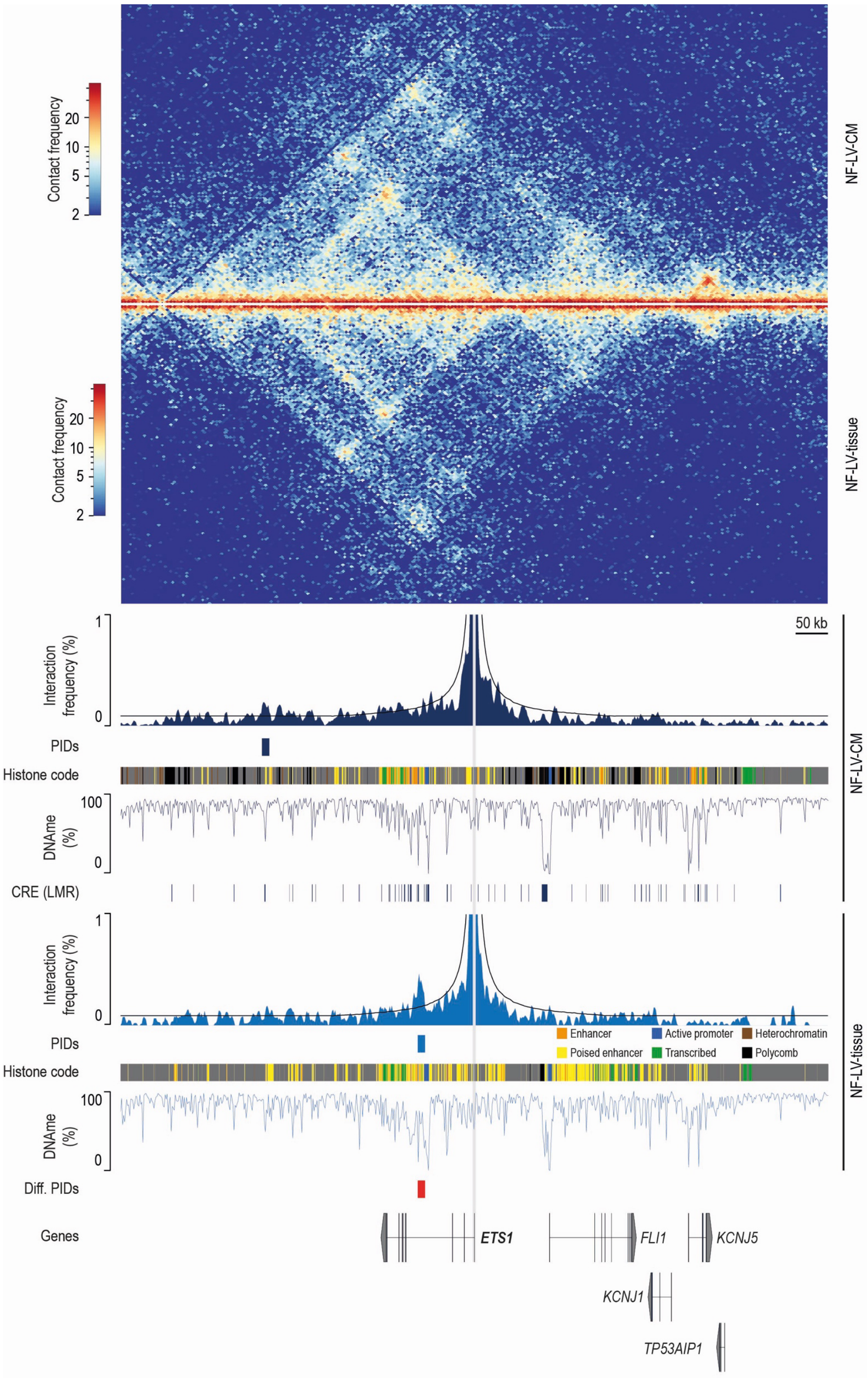

Supplementary Fig. 8: Comparison of cardiac and CM chromatin interactions for the *ETS1* locus.
see legend Supplementary Fig. 3

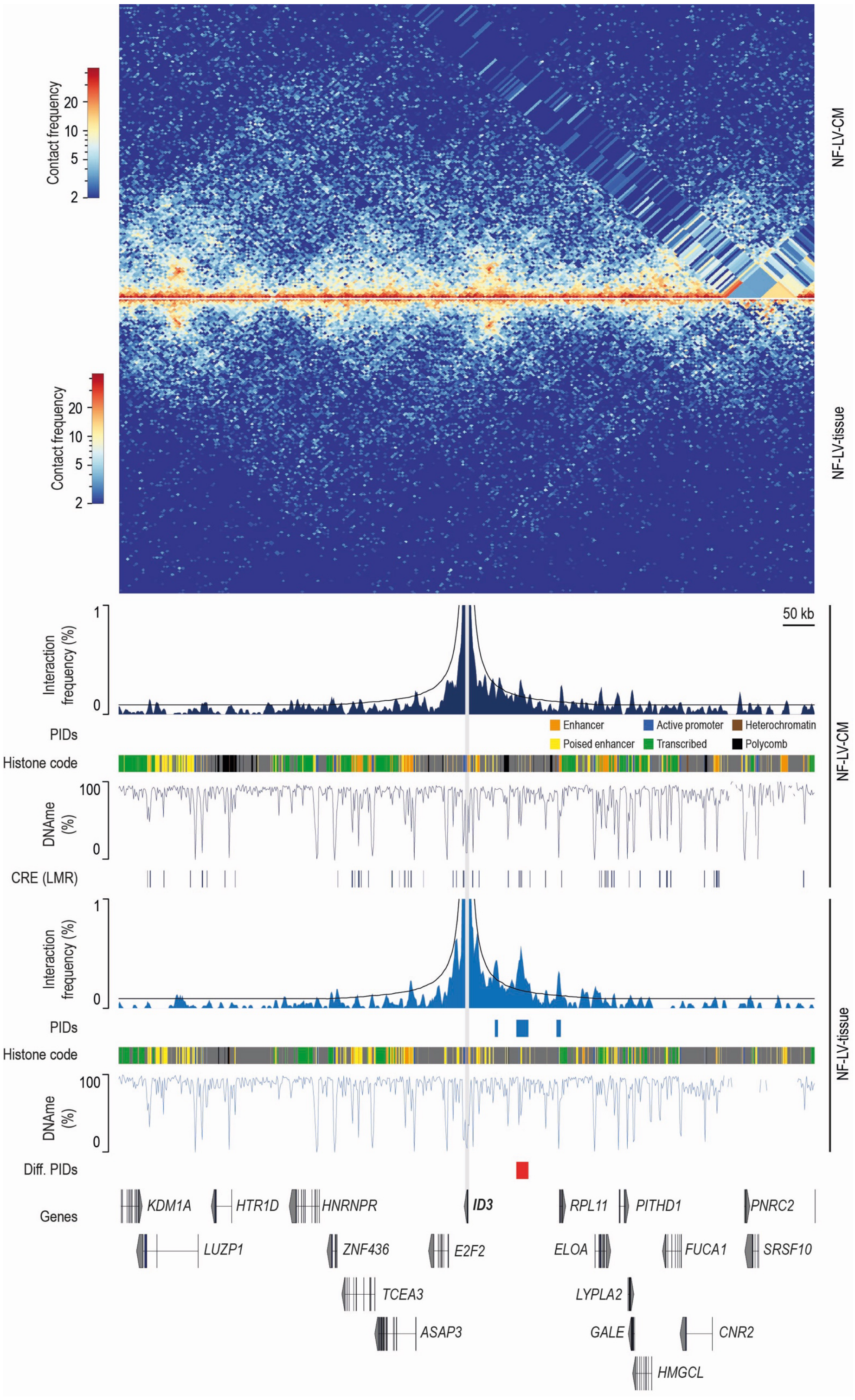

Supplementary Fig. 9: Comparison of cardiac and CM chromatin interactions for the *ID3* locus.
see legend Supplementary Fig. 3

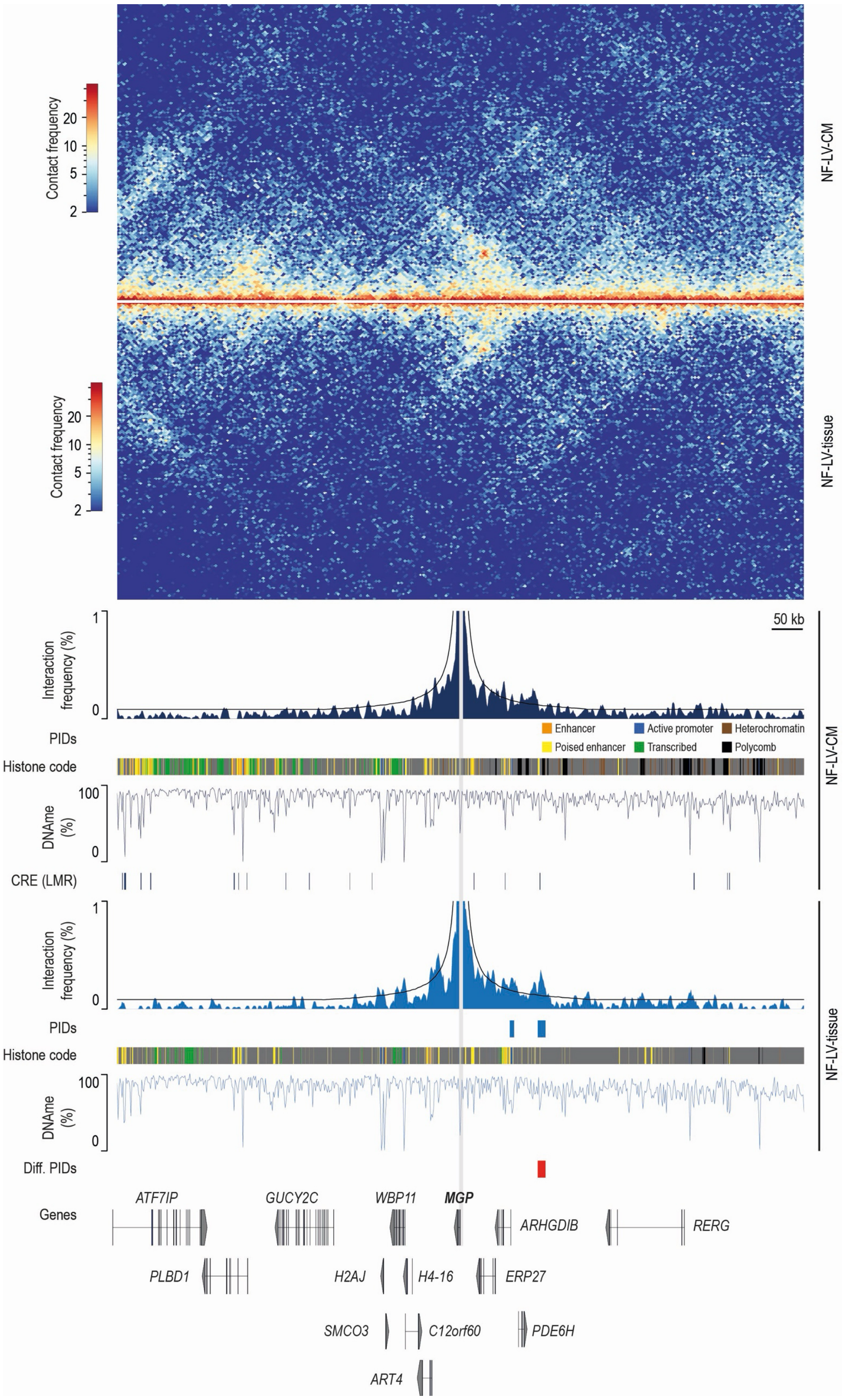

Supplementary Fig. 10: Comparison of cardiac and CM chromatin interactions for the *MGP* locus.
see legend Supplementary Fig. 3

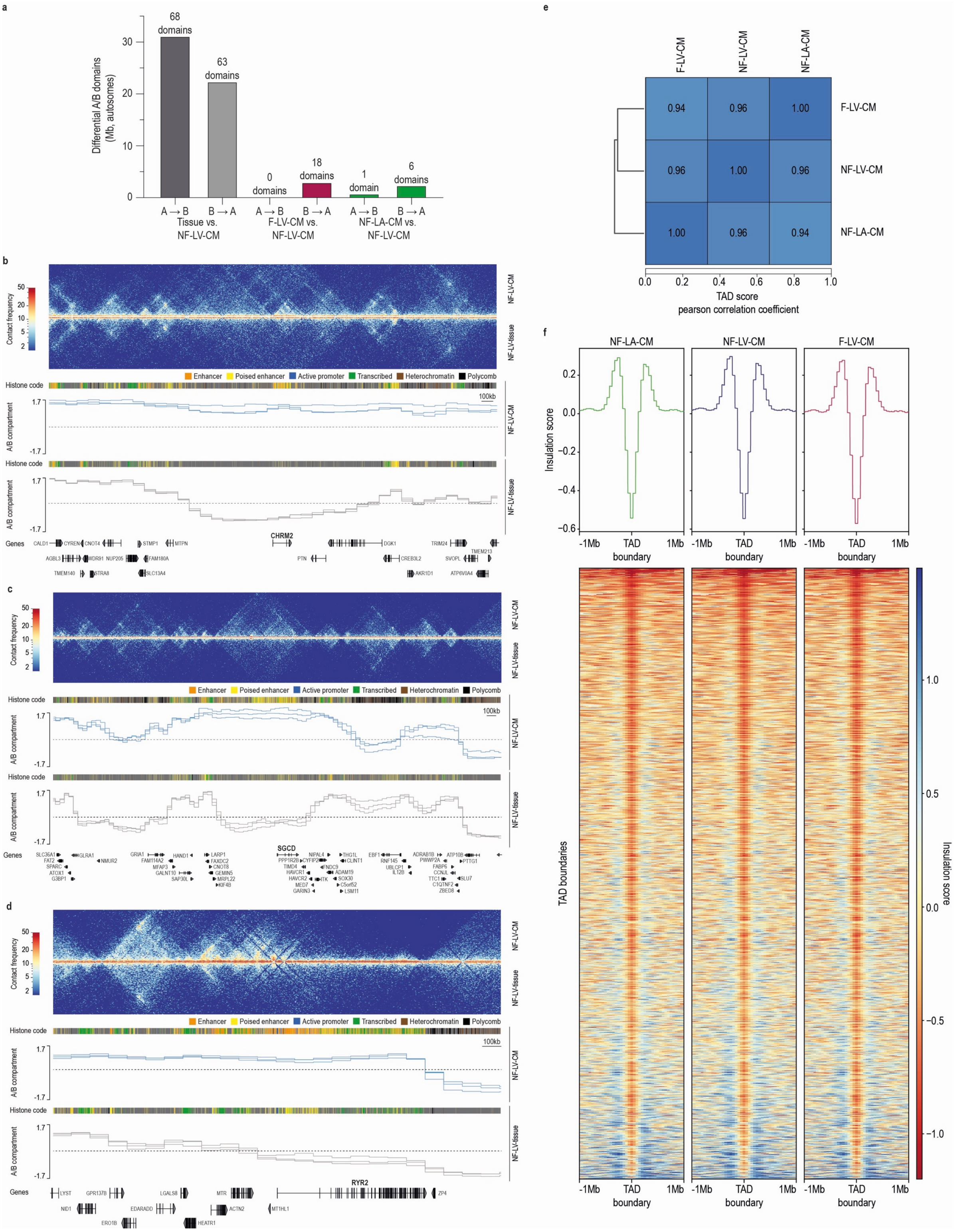

Supplementary Fig. 11: Higher-order chromatin structure of bulk cardiac nuclei, NF-LV-CM, NF-LA-CM, and F-LV-CM.

a Bar plot showing the number and the sequence length of significant A/B compartment switches (MB).

b, **c**, **d** A/B compartment switches at loci harboring *CHRM2, SGCD, and RYR2,* respectively. Shown are original traces of chromatin interaction frequency heatmap at 5 kb resolution (Hi-C), chromatin state annotation tracks, and original traces of PCA analysis values corresponding to A/B compartments. A/B and Hi-C data shown were derived from three biological replicates.

e, f Hi-C data were derived from 9 (NF-LV-CM), 6 (NF-LA-CM), and 8 (F-LV-CM) biological replicates. Heatmap of Pearson correlation coefficients of TAD separation scores (**e**). Average plots and heatmaps of TAD separation scores are shown for TAD boundaries of NF-LV-CM across the different CM subtypes (**f**).

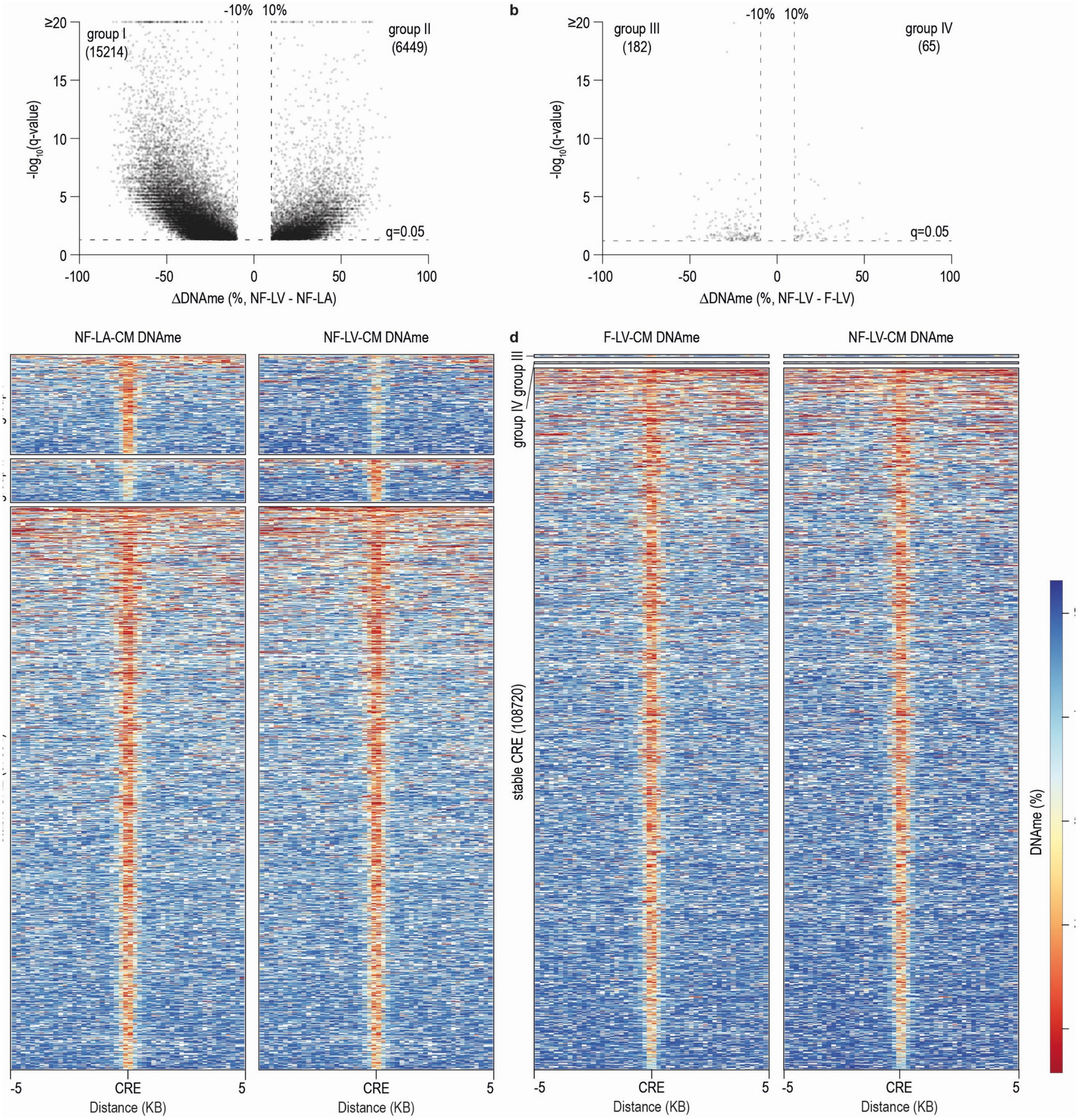

Supplementary Fig. 12: Differential CRE analysis of NF-LA-CM and F-LV-CM compared to NF-LV-CM.
a, b Volcano plots highlight *Cis*-regulatory elements grouped by differential methylation and condition (LMR-I to IV) in NF-LA-CM (**a**), and F-LV-CM (**b**) compared to NF-LV-CM. Cut-off criteria ($q<0.05$ and Δ10%) are highlighted, and the number of CREs meeting the criteria is given.
c, d Heatmaps of mCpG are shown for stable and differential CREs for NF-LV-CM, NF-LA-CM, and F-LV-CM. Plotted groups correspond to panels (**a** and **b**). Data were derived from 4 biological replicates (**a-d**).

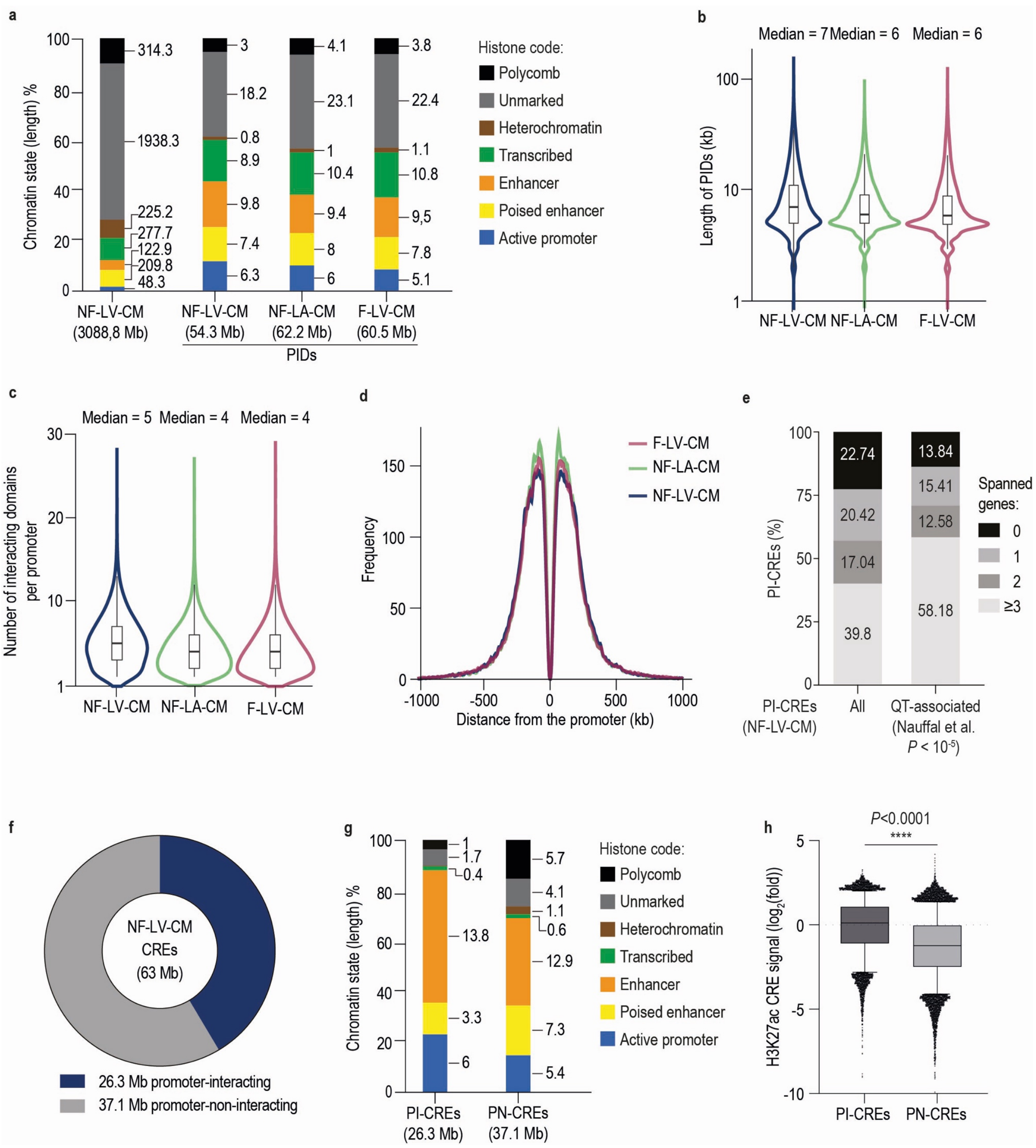

Supplementary Fig. 13: Characterization of PIDs, PI-CREs and PN-CREs.

a Percentage of histone codes identified using ChromHMM in the human genome and PIDs. Data shown as sequence length.

b,c Violin and box plots of length (b) and count (c) of PIDs (Promoter-interacting domains) of NF-LV-CM, NF-LA-CM, and F-LV-CM.

d Histogram representing distance of PIDs to their associated promoter.

e Percentage CRE-promoter interactions spanning reference genes. Data is shown for all detected PI-CREs and those associated with QT-associated genetic variants of CREs according to Naufal et al.[1] applying sub-threshold statistical criteria.

f Pie chart showing the overlap between CREs (LMRs) and PIDs of NF-LV-CM.

g Percentage of histone codes identified using ChromHMM in PI-CREs (Promoter-interacting CREs) and PN-CREs (Promoter-non-interacting CREs) (**f**). Data shown as sequence length.

h Box and Whiskers plot (5-95 percentile) of H3K27ac signal of PI-CREs and PN-CREs in NF-LV-CM. ****P < 0.0001; Two-tailed Mann-Whitney test.

a-h Hi-C data were derived from 9 (NF-LV-CM), 6 (NF-LA-CM), and 8 (F-LV-CM) biological replicates.

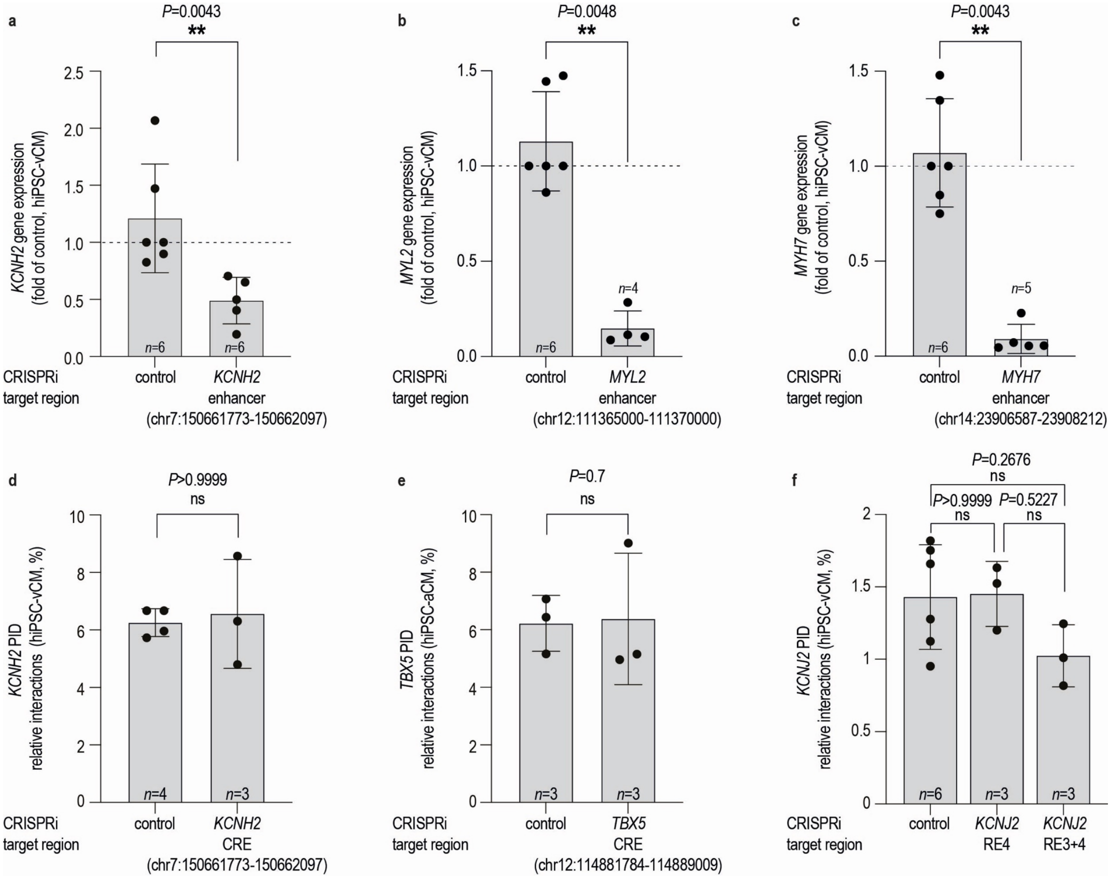

Supplementary Fig. 14: Functional silencing of promoter-interacting CREs and its impact on interaction strength.

a-c AAV-mediated CRISPRi silencing of regulatory elements of *KCNH2* **(a)**, *MYL2* **(b)**, and *MYH7* **(c)**. Targeted genomic regions are indicated. Gene expression was quantified by RT-qPCR. Data is presented as mean ± SD. *n*-numbers indicated on the plot represent independent biological replicates. $^{**}P < 0.01$; Two-tailed Mann-Whitney test.

d-f Relative PID-promoter interaction frequency upon AAV-mediated CRISPRi silencing of PI-CREs of *KCNH2* **(d)**, *TBX5* **(e)**, *KCNJ2* **(f)**. Corresponding gene expression data for these CRISPRi experiments are shown in panel a, Fig. 4d, and Fig. 8b, respectively. Data is presented as mean ± SD. *n*-numbers indicated on the plot represent independent biological replicates. ns, $P > 0.05$; Two-tailed Mann-Whitney test (**d, e**), and Kruskal-Wallis test with Dunn's adjustment for multiple comparisons (**f**).

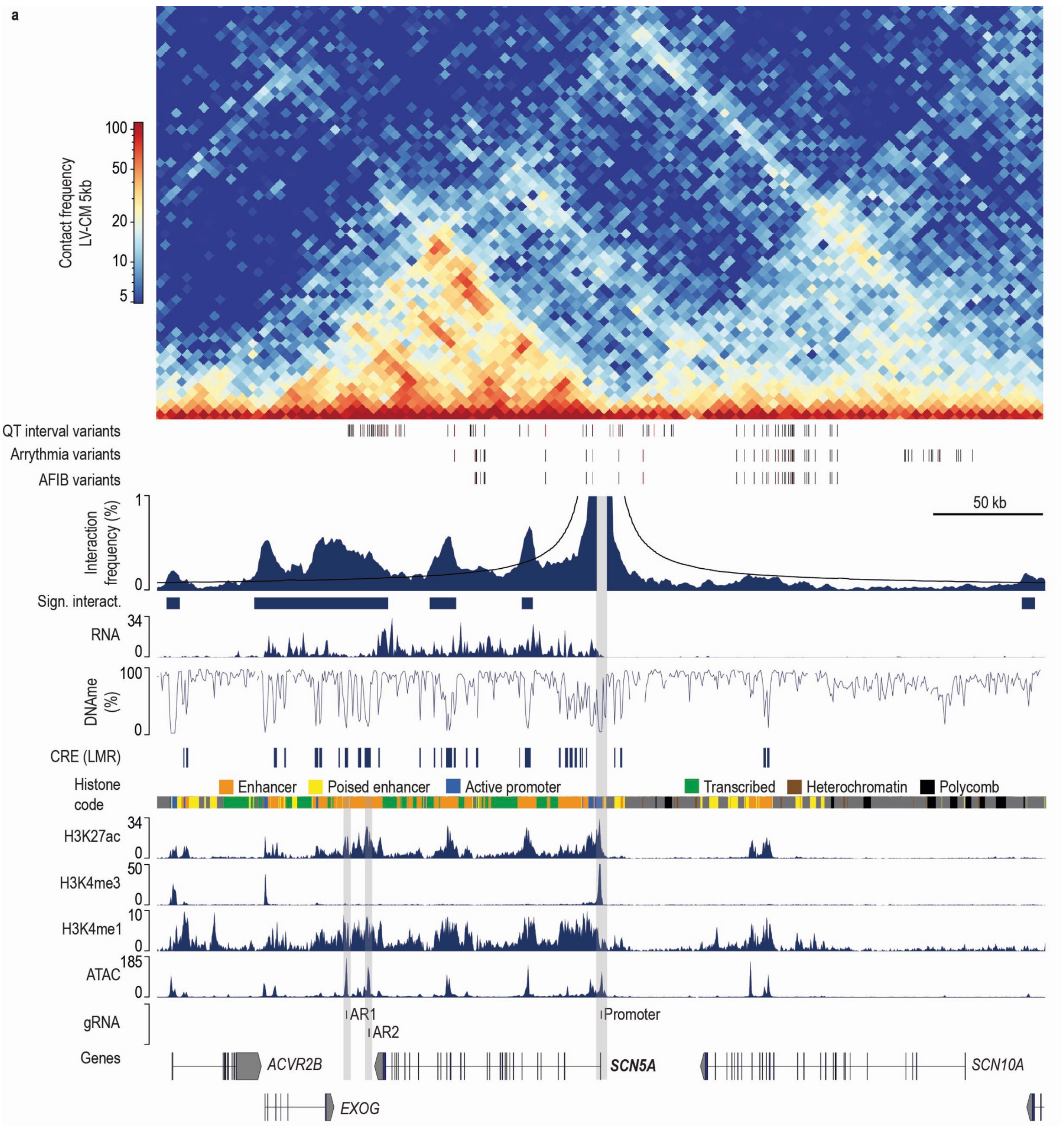

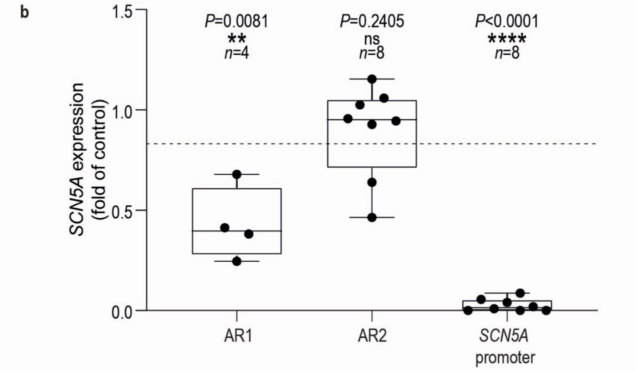

Supplementary Fig. 15: Functional relevance of disease-associated PI-CREs in locus containing the *SCN5A* gene.
a Heatmap of chromatin interactions (5 kb resolution) and GWAS variants for long QT syndrome, cardiac arrhythmia, and AFIB (lead-SNPs = red, proxy-SNPs = black). Virtual viewpoint analysis for the *SCN5A* promoter (1 kb resolution) and chromatin states are shown. Original traces for gene expression (RNA-seq), chromatin accessibility (ATAC-seq), and H3K27ac (ChIP-seq) of NF-LV-CM for the genomic locus containing *SCN5A* are depicted. CREs and PIDs are annotated. Hi-C data derived from 9 biological replicates. Grey areas highlight CRISPRi-silenced regions.
b CRISPRi silencing of two *SCN5A* promoter interacting elements (AR1 and AR2) and the *SCN5A* promoter. Lenti-transduced hiPSC-vCM were FACS-sorted, and gene expression was measured by RT-qPCR. *SCN5A* gene expression values are shown as box and whiskers (box=25th-75th percentile, line=median, whiskers= minimum to maximum). *n*-numbers indicated on the plot represent independent biological replicates. ns, $P \geq 0.05$; $^{**}P < 0.01$; $^{****}P < 0.0001$; Two-tailed one sample t-test.

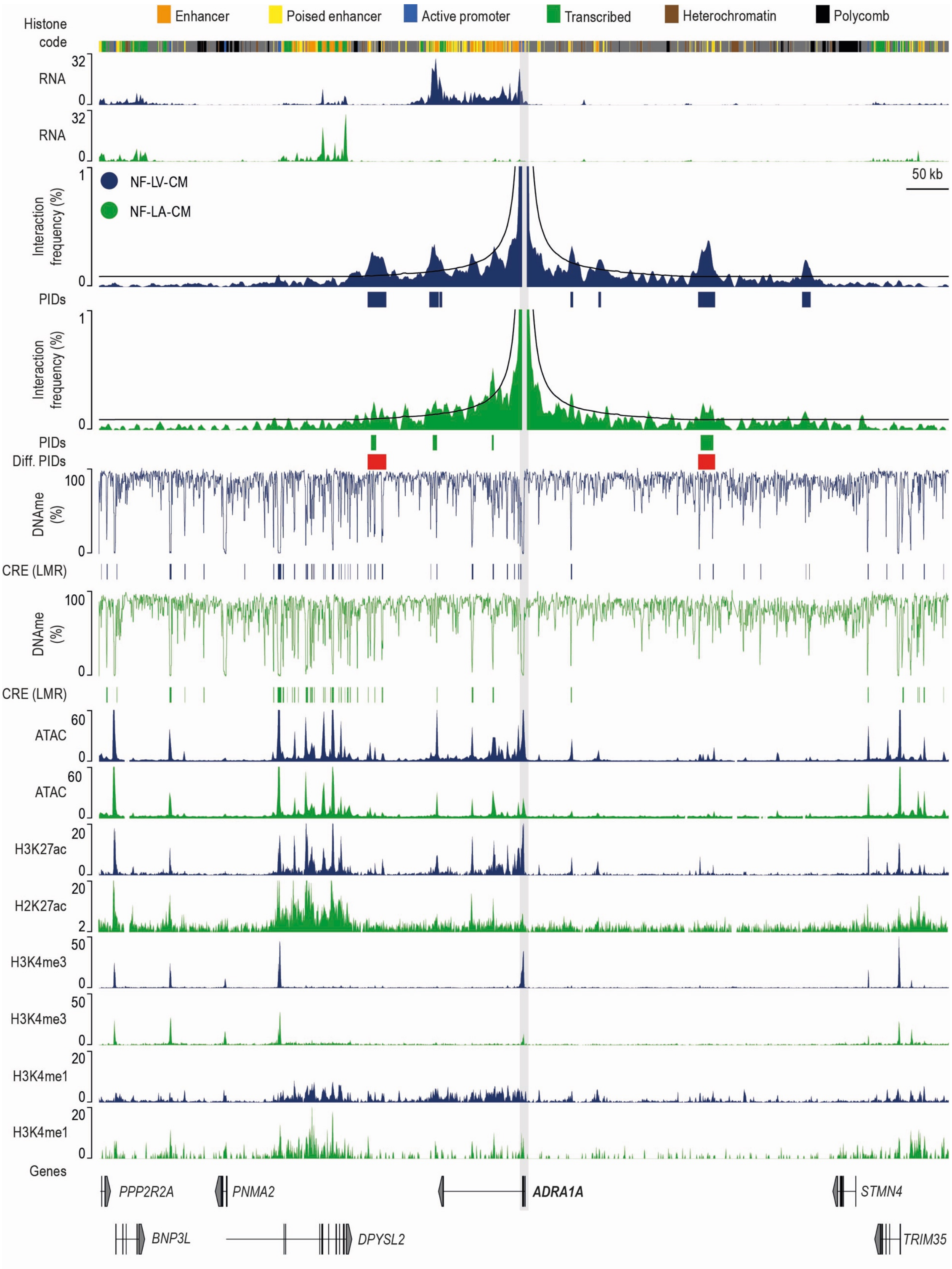

Supplementary Fig. 16: Comparison of atrial and ventricular CM chromatin interactions of the *ADRA1A* promoter.

Original traces of and histone marks (ChromHMM), virtual viewpoint analysis (1 kb resolution) of *ADRA1A* promoter interactions (Hi-C), mCpG (DNA methylation sequencing), gene expression (RNA-seq), chromatin accessibility (ATAC-seq), and histone modification (ChIP-seq). CREs, PIDs, and differential PIDs are annotated. NF-LV-CM and NF-LA-CM tracks are shown in blue and green, respectively. Grey area highlights the *ADRA1A* promoter. Hi-C data derived from *n* biological replicates: NF-LV-CM, 9; NF-LA-CM, 6.

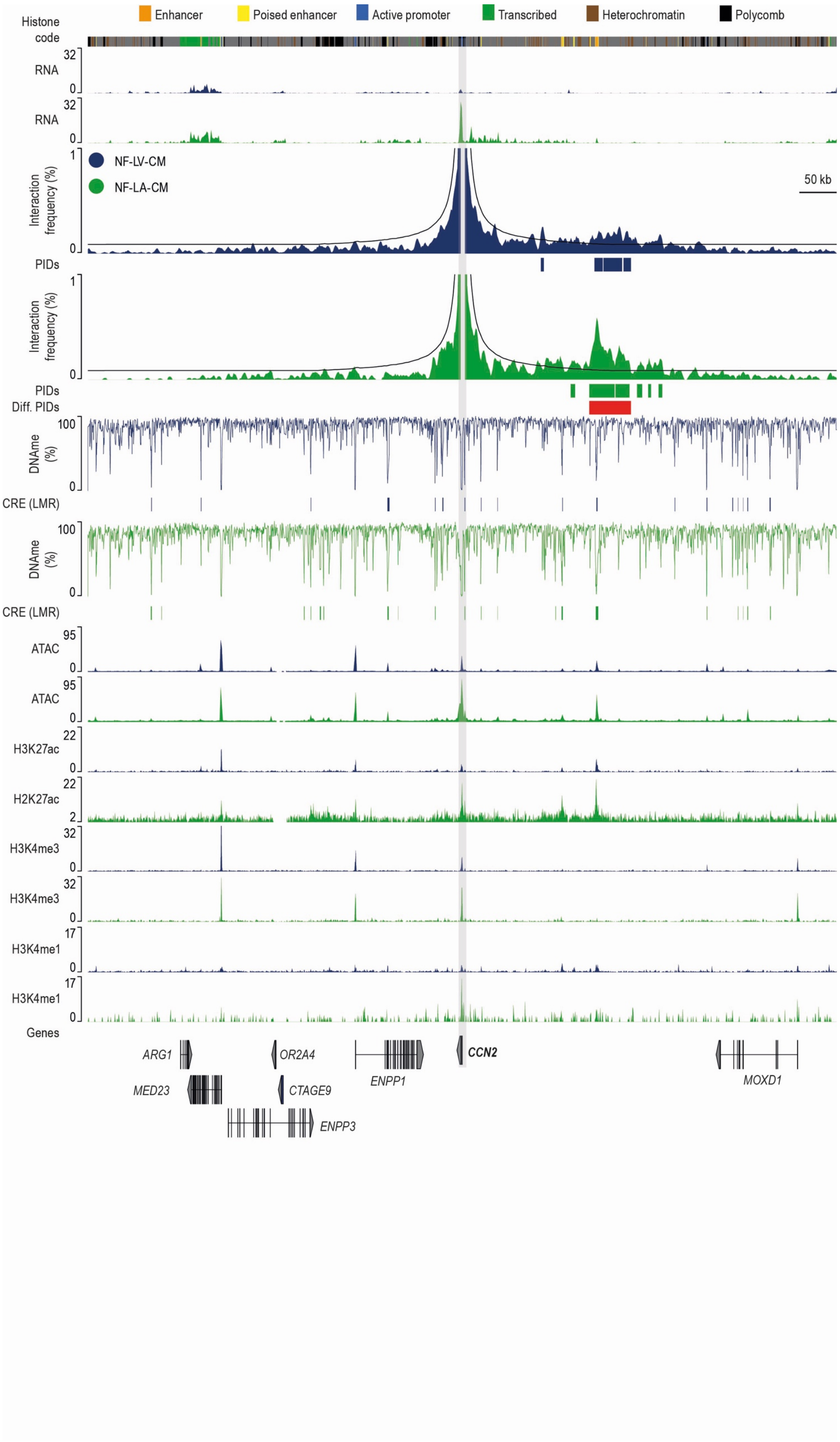

Supplementary Fig. 17: Comparison of atrial and ventricular CM chromatin interactions of the *CCN2* promoter.
see legend Supplementary Fig. 16

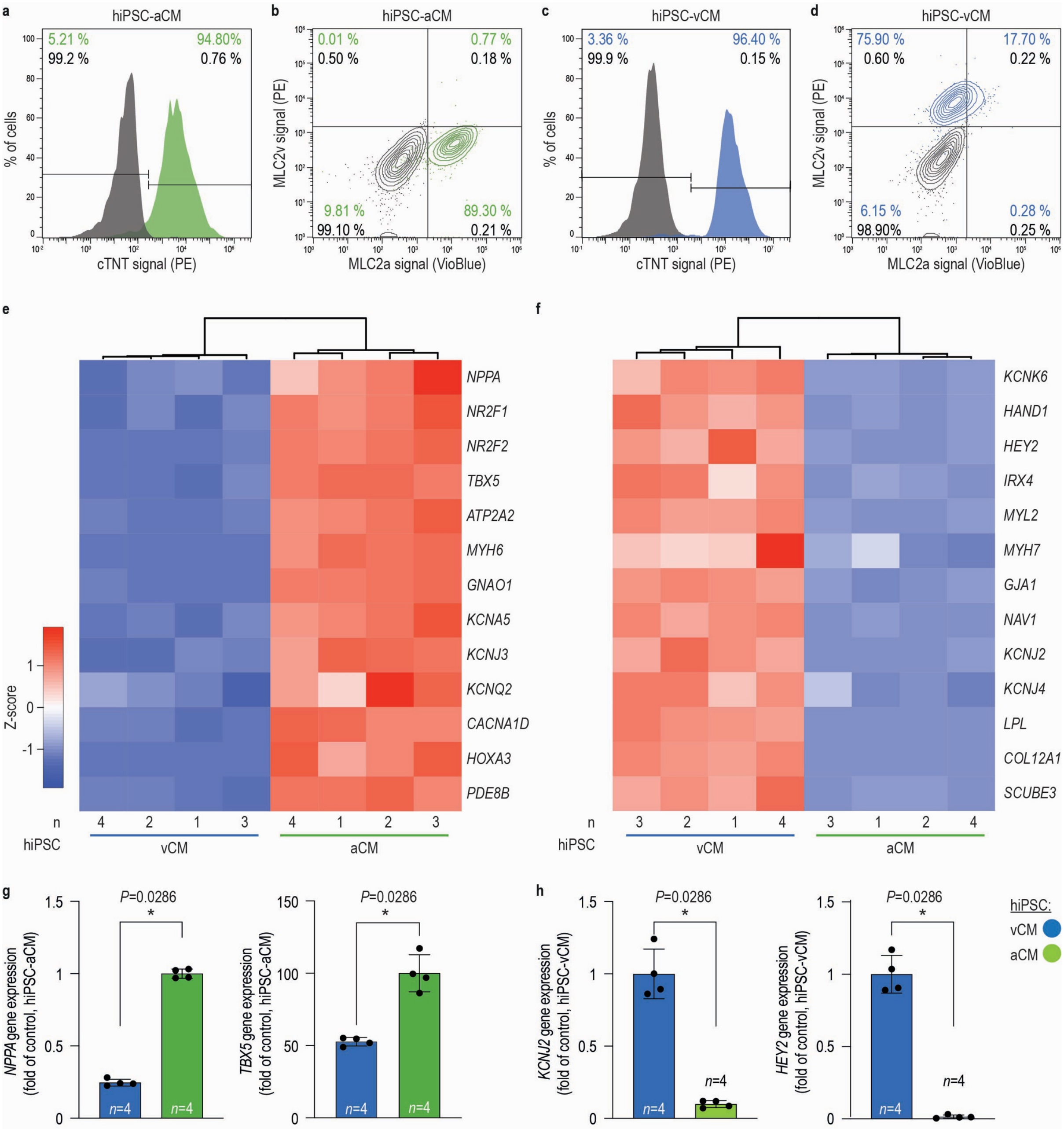

Supplementary Fig. 18: Characterization of hiPSC-aCM and hiPSC-vCM.

a-d Flow cytometric analysis of marker genes characteristic for CM (cTNT) as well as for atrial (MLC2a, *MYL7*) and ventricular (MLC2v, *MYL2*) CM subtypes. Shown are representative data for hiPSC-aCM (green) and hiPSC-vCM (red) stained with fluorescently labeled antibodies and the respective staining controls (grey). The relative cell numbers (%) are displayed using the matching color code.

e-h RNA-seq gene expression analysis of hiPSC-aCM and hiPSC-vCM. Heatmaps display z-score transformed gene expression data of atrial **(e)** and ventricular **(f)** marker genes. Columns were hierarchically clustered. Bar plots of mean ± SD **(g,h)** highlight relative expression data for genes studied in this manuscript. *n*=4 biological replicates, $^*P < 0.05$; Two-tailed Mann-Whitney test.

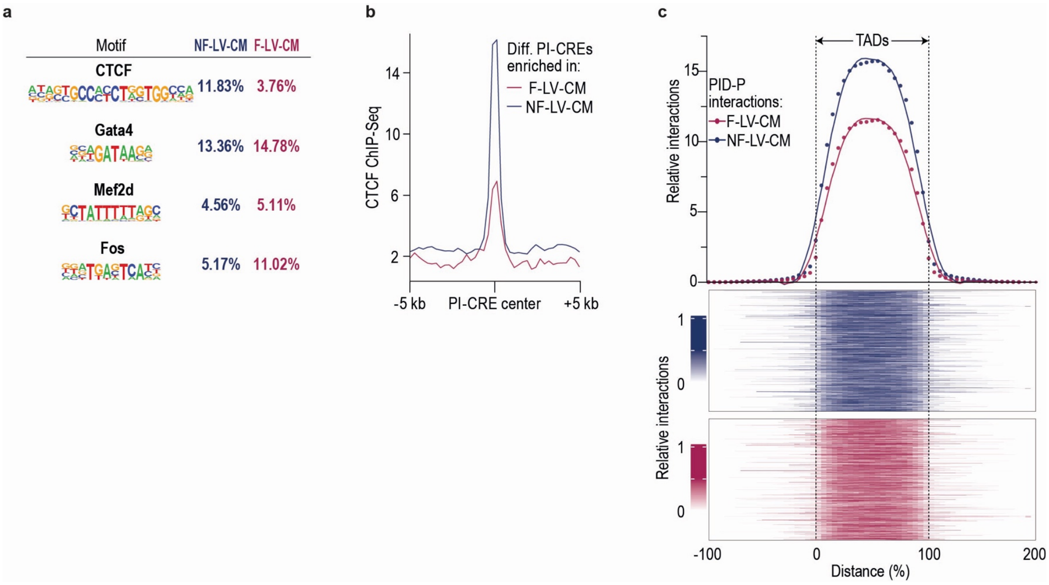

Supplementary Fig. 19: Comparison of chromatin interactions in non-failing and failing cardiomyocytes of the left ventricle.

a Transcription factor motif enrichment (%) in differential PI-CREs.

b Enrichment plot of CTCF is shown at differential PI-CREs between NF-LV-CM and F-LV-CM.

c Characterization of PID-promoter interaction localization relative to topologically associating domains (TADs). The upper plot shows the sum of relative interactions. The lower heatmap represents the relative interaction in individual TADs. The intensity of blue and red horizontal lines represents the fraction of distal element-promoter interactions spanning the TAD locus in NF-LV-CM and F-LV-CM, respectively.

a-c Hi-C data were derived from 9 (NF-LV-CM), and 8 (F-LV-CM) biological replicates.

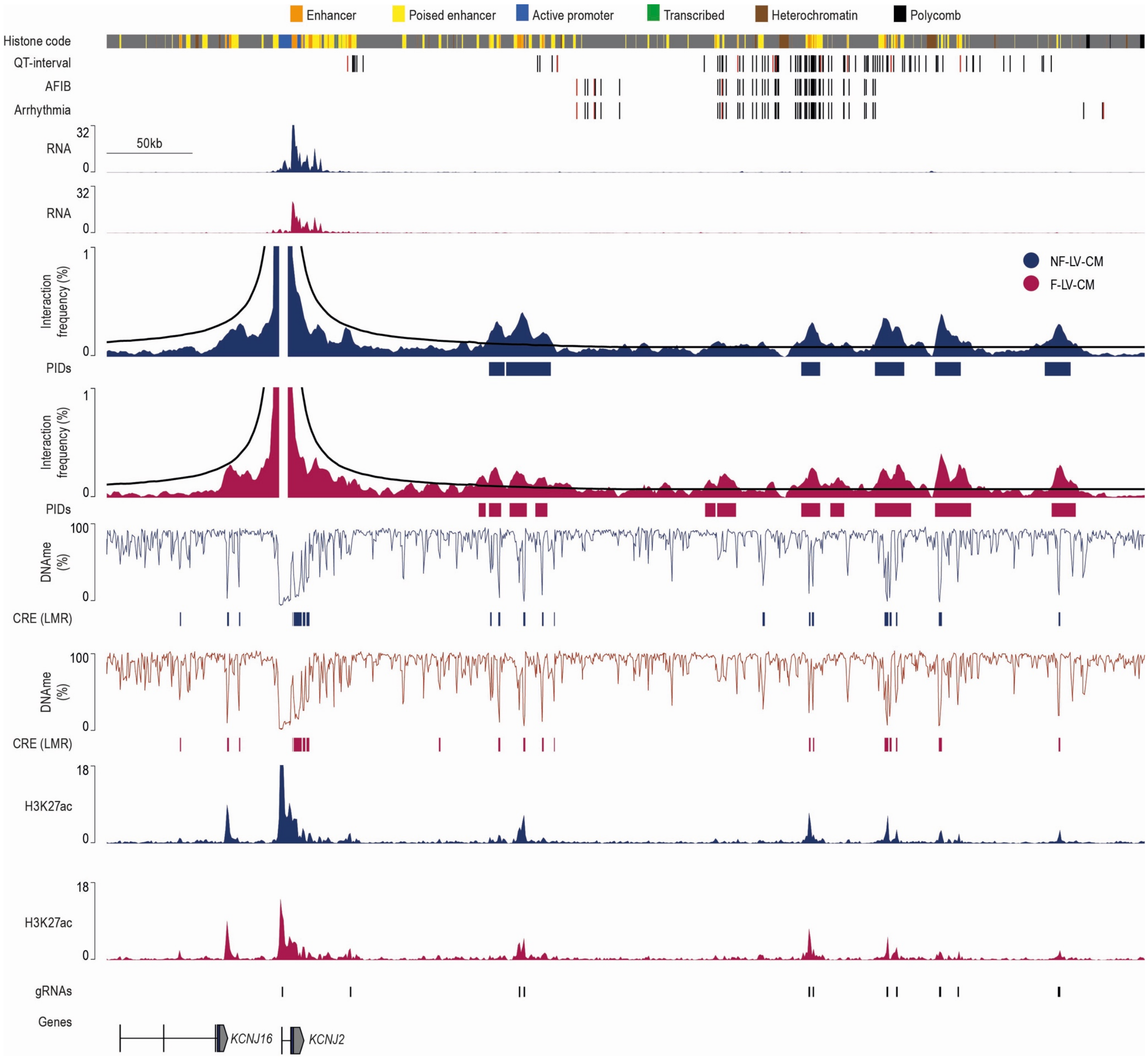

Supplementary Fig. 20: Comparison of non-failing and failing ventricular CM chromatin interactions for the *KCNJ2* promoter.

Virtual viewpoint analysis of *KCNJ2* promoter interactions (1 kb resolution), gene expression (RNA-seq), H3K27ac (ChIP-seq), and histone code (ChromHMM) are shown. CRE and PIDs are annotated. NF-LV-CM and F-LV-CM tracks are shown in blue and red, respectively. Hi-C data were derived from 9 (NF-LV-CM) and 8 (F-LV-CM) biological replicates.

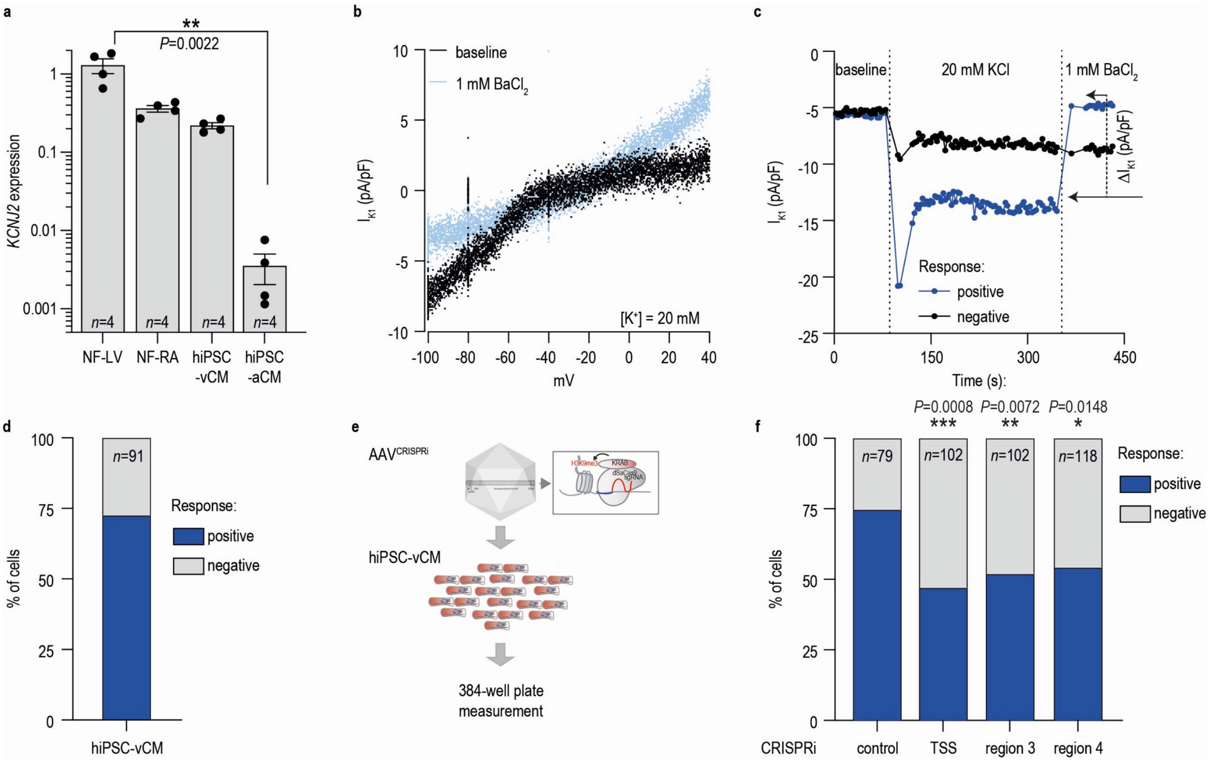

Supplementary Fig. 21: *KCNJ2* expression and electrophysiological assessment of I_{K1} in hiPSC-vCM using automated patch clamp.

a *KCNJ2* expression in non-failing ventricular tissue, non-failing atrial cardiac tissue, hiPSC-vCM and hiPSC-aCM. Gene expression was measured by RT-qPCR (n = 4). $^{**}P < 0.01$; Kruskal-Wallis test with Dunn's correction for multiple comparisons. Data is presented as mean ± SD. *n*-numbers indicated on the plot represent independent biological replicates.

b Representative traces of hiPSC-vCM showing basal inward rectifier current (I_{K1}) in the presence of 20 mM KCl (black) and $BaCl_2$ (blue), respectively. Currents were activated with a ramp pulse from −100 to +40 mV at 0.5 Hz.
c Representative traces of I_{K1} time course demonstrating cells with positive (blue) and negative (black) response to 20 mM KCl and $BaCl_2$.
d Percent of hiPSC-vCM responsive or non-responsive to 20 mM KCl and $BaCl_2$.
e Scheme of high-throughput patch clamp assay of hiPSC-vCM with AAV-CRISPRi treatment.
f Distribution of positive and negative responses in hiPSC-vCM after application of 20 mM KCl and $BaCl_2$, compared using Chi-square test. *P* values were corrected for multiple comparisons according to the Bonferroni correction. n = number of cells from three batches. $^{*}P < 0.05$; $^{**}P < 0.01$; $^{***}P < 0.001$

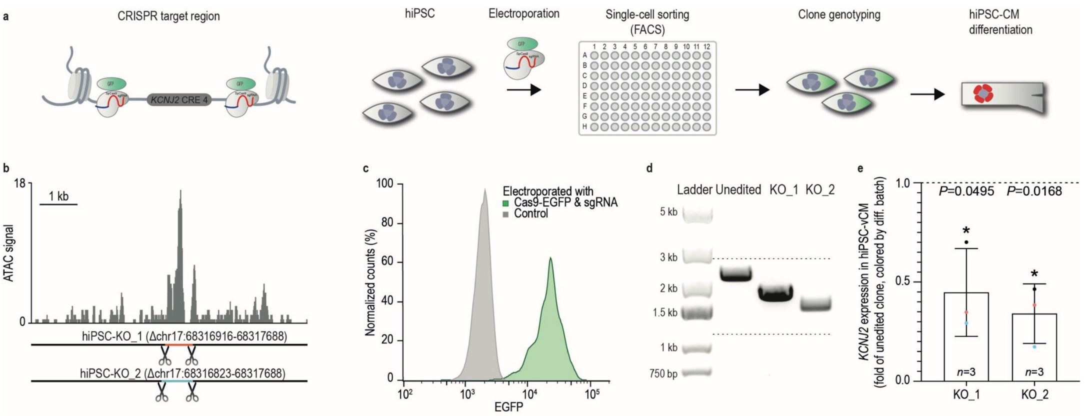

Supplementary Fig. 22: CRISPR-mediated deletion of *KCNJ2* RE4 in hiPSC and validation in the derived CM.

a Schematic overview of the experimental workflow. Dual sgRNAs flanking the *KCNJ2* CRE (region 4) were co-electroporated with Cas9-EGFP into hiPSC. EGFP-positive single cells were FACS-sorted into 96-well plates, clonally expanded, and genotyped to confirm RE4 deletion. Successfully edited hiPSC clones were subsequently differentiated into hiPSC-vCM.

b Chromatin accessibility (ATAC-seq) at the *KCNJ2* RE4 locus. The deleted RE4 region of two independent clones is indicated below and flanked by scissors.

c Histogram showing flow cytometry analysis in electroporated hiPSCs (green) overlayed onto untransfected control cells (grey).

d Genotyping of unedited and knockout clones. Agarose gel electrophoresis of amplicons using primers flanking the *KCNJ2* RE4, showing successful deletion in hiPSC-KO_1 and hiPSC-KO_2 clones, as compared to unedited clone.

e *KCNJ2* expression levels ventricular CM derived from hiPSC-KO_1 and hiPSC-KO_2, assessed using RT-qPCR. Data is presented as mean ± SD. three independent differentiation batches were used. *, $P < 0.05$; One-sample Wilcoxon test.

References

1 Nauffal, V. *et al.* Monogenic and Polygenic Contributions to QTc Prolongation in the Population. *Circulation* **145**, 1524-1533 (2022). https://doi.org/10.1161/CIRCULATIONAHA.121.057261
