# Supplementary material for: Chamber-specific chromatin architecture guides functional interpretation of disease-associated Cis-regulatory elements in human cardiomyocytes
Source: Nat Commun. 2026 Jan 12;17:117. doi: 10.1038/s41467-025-67220-7 (PMC12796357; doi:10.1038/s41467-025-67220-7)
Supplement: Supplementary file 2 — Description of Additional Supplementary Files [file 41467_2025_67220_MOESM2_ESM.pdf]

Description of Additional Supplementary Files

Title: Supp Data 1

Description: Cardiomyocyte-marker genes and housekeeping genes used in Fig. 2

Title: Supp Data 2

Description: Sample characteristics

Title: Supp Data 3

Description: Detailed Hi-C read statistics

Title: Supp Data 4

Description: PIDs in NF-LV-CM

Title: Supp Data 5

Description: PIDs in NF-LA-CM

Title: Supp Data 6

Description: PIDs in F-LV-CM

Title: Supp Data 7

Description: PI-CREs in NF-LV-CM

Title: Supp Data 8

Description: PI-CREs in NF-LA-CM

Title: Supp Data 9

Description: PI-CREs in F-LV-CM

Title: Supp Data 10

Description: Oligo sequences
